# Matrix metalloproteinase activity in the lung is increased in Hermansky-Pudlak syndrome

**DOI:** 10.1186/s13023-019-1143-0

**Published:** 2019-07-04

**Authors:** Ross Summer, Rachana Krishna, DeLeila Schriner, Karina Cuevas-Mora, Dominic Sales, Rachel Para, Jesse Roman, Carl Nieweld, Bernadette R. Gochuico, Freddy Romero

**Affiliations:** 1 Center for Translational Medicine and Jane and Leonard Korman Lung Center, Thomas Jefferson University, Philadelphia, USA; 2 Medical Genetics Branch, National Human Genome Research Institute, National Institutes of Health, Bethesda, MD USA; 3 Center for Translational Medicine and Jane and Leonard Korman Respiratory Institute, Thomas Jefferson University, 1020 Locust Street, JAH 354, Philadelphia, PA 19107 USA

**Keywords:** Hermansky-Pudlak syndrome, Matrix metalloproteinase, Pulmonary fibrosis

## Abstract

**Background:**

Hermansky-Pudlak syndrome (HPS) is a rare autosomal recessive disorder characterized by oculocutaneous albinism and platelet dysfunction and can sometimes lead to a highly aggressive form of pulmonary fibrosis that mimics the fatal lung condition called idiopathic pulmonary fibrosis (IPF). Although the activities of various matrix metalloproteinases (MMPs) are known to be dysregulated in IPF, it remains to be determined whether similar changes in these enzymes can be detected in HPS.

**Results:**

Here, we show that transcript and protein levels as well as enzymatic activities of MMP-2 and -9 are markedly increased in the lungs of mice carrying the HPS Ap3b1 gene mutation. Moreover, immunohistochemical staining localized this increase in MMP expression to the distal pulmonary epithelium, and shRNA knockdown of the Ap3b1 gene in cultured lung epithelial cells resulted in a similar upregulation in MMP-2 and -9 expression. Mechanistically, we found that upregulation in MMP expression associated with increased activity of the serine/threonine kinase Akt, and pharmacological inhibition of this enzyme resulted in a dramatic suppression of MMP expression in Ap3b1 deficient lung epithelial cells. Similarly, levels and activity of different MMPs were also found to be increased in the lungs of mice carrying the Bloc3 HPS gene mutation and in the bronchoalveolar lavage fluid of subjects with HPS. However, an association between MMP activity and disease severity was not detected in these individuals.

**Conclusions:**

In summary, our findings indicate that MMP activity is dysregulated in the HPS lung, suggesting a role for these proteases as biological markers or pathogenic players in HPS lung disease.

**Electronic supplementary material:**

The online version of this article (10.1186/s13023-019-1143-0) contains supplementary material, which is available to authorized users.

## Background

Hermansky–Pudlak syndrome (HPS) represents a heterogeneous group of rare autosomal recessive disorders characterized by oculocutaneous albinism, platelet dysfunction and, in some cases, pulmonary fibrosis [1, 2]. To date, ten different HPS genes have been identified, and each encodes for different proteins involved in the biogenesis or trafficking of lysosomes or lysosome-related organelles [2–5]. Although each HPS mutation has been shown to affect pigment and platelet functions, only those genes associated with the HPS-1, HPS-2, or HPS-4 genetic subtypes are linked to pulmonary fibrosis [1, 4]. Importantly, in these individuals the development of pulmonary fibrosis is often a fatal complication, leading to death within just several years of its initial detection.

Matrix metalloproteinases (MMPs) are a family of zinc-dependent proteolytic enzymes that are best known for their role in degrading extracellular matrix proteins, although are also responsible for activating or inhibiting a wide range of other effector molecules [6, 7]. Dysregulated MMP activity has been linked to the pathogenesis of numerous chronic lung diseases, including asthma, emphysema, cystic fibrosis, and fibrotic lung diseases such as idiopathic pulmonary fibrosis (IPF) [7–11]. Although it remains unclear how alterations in this group of enzymes can lead to such diverse lung pathologies, it has been suggested that differences in the expression of enzymatic subtypes might play a contributory role [7–11]. Indeed, levels of specific MMPs have been shown to be elevated in the blood and bronchoalvelolar lavage fluid (BALF) of patients with IPF [10–13], including MMP-2, MMP-7 and MMP-9. While these changes were originally thought to be important for limiting the severity of fibrotic remodeling (by degrading extracellular matrix proteins), targeted deletion of individual MMP genes in mice has yielded mixed results [10, 14–16], emphasizing the complex biology of MMPs in the lung.

MMP activity is under strict regulation by a variety of mechanisms at the transcriptional and post-translational levels. Recent work in several model systems has demonstrated the importance of the phosphatidylinositol 3-kinase (PI3K)-Akt pathway in the regulation of MMPs [17–19]. Akt is a serine/threonine kinase, which controls a wide range of biological processes typified in fibrotic tissues, including those intimately involved in growth, proliferation, migration, and metabolic reprogramming of fibroblasts [20, 21]. Additionally, Akt activity has also been shown to be upregulated in type II alveolar epithelial cells (AEC2) of patients with IPF, and inhibition of enzymatic activity in these cells has proven to be effective in reducing tissue remodeling to bleomycin in the mouse lung [21].

In this study, we employed in vitro and in vivo model systems as well as utilized mouse and human tissues to establish whether levels or activities of MMPs were altered in the HPS lung and to determine whether these changes occurred before or after the onset of pulmonary fibrosis.

## Methods

### Animals

Wild-type, HPS1, and HPS2 mice (C57B/6 J, 8–10 weeks old) were purchased from the Jackson Laboratory (Bar Harbor, ME) and housed in a pathogen-free animal facility at Thomas Jefferson University. HPS1 mice have homozygous mutation of the *Hps1* gene, which encodes for a protein called BLOC-3, and HPS2 mice have homozygous mutation in the adaptor protein 3b1 (*Ap3b1)* gene, which is a subunit of the AP-3 protein complex. In general, HPS mice are phenotypically normal, except for a light coat appearance. HPS 1 and 2 mice also have large lamellar bodies in the alveolar epithelial type II cells of their lungs. Both strains of mice are also exquisitely sensitive to bleomycin. Throughout the study period, wild-type and HPS mice were maintained on a standard chow diet (13.5% calories from fat, 58% from carbohydrates, and 28.5% from protein) and permitted to feed ad libitum. Prior to the initiation of any study, the Institutional Animal Care and Use Committee at Thomas Jefferson University approved all animal protocols.

### Human subjects

The diagnosis of HPS was established based on published criteria [22, 23]. Healthy controls were individuals without any known lung disease. Age, gender and smoking history for subjects are listed in Additional file 1: Table S1. All patients provided written informed consent to protocols 95-HG-0193 (clinicaltrials.gov NCT00001456) and 04-HG-0211 (clinicaltrials.gov NCT00084305). All study protocols were approved by the Institutional Review Boards at Thomas Jefferson University and the National Human Genome Research Institute prior to the initiation of any studies. BAL was performed and samples were processed as previously described [24].

### Bleomycin-induced lung injury

Lung injury was induced by instilling 0.025 U of bleomycin into the posterior oropharynx of anesthetized mice. Because HPS1 and HPS2 mice are more sensitive to bleomycin, lower doses of bleomycin were required for these investigations [2, 25, 26].

### Measurement of MMP-2 and MMP-9 activity

The activity of MMP-2 and -9 was assessed by gelatin zymography as previously described [8, 27]. Protein concentration was determined by Pierce™ BCA assay kit (Thermo Scientific, Rockford, IL). Murine and human BALF and lung homogenates were separated by electrophoresis using 10% SDS-polyacrylamide gels containing 0.1% gelatin. Gels were then washed in 2.5% triton 100 renaturing buffer followed by overnight incubation in developing buffer. To visualize bands gels were stained with 0.5% Coomassie blue for 1 h and then destained with 40% methanol/10% acetic acid until clear bands were visualized. Densitometry was performed as previously described and the MMPs activity were normalized for total BALF and lung homogenates protein concentration.

### Lung histology

Lungs were removed *en bloc* and immersed in fixative at 4 °C for 18 h. Tissues underwent a series of dehydration steps prior to being embedded in paraffin. Prior to performing immunohistochemical staining we performed antigen retrieval and quenched endogenous peroxidases. Primary antibodies to MMP-2 (Abcam, Cambridge, UK) and MMP-9 (Thermo Scientific, Rockford, IL) were used in our studies. To visualize antibody binding, sections were exposed to Vectastain ABC (Vector Laboratories, Burlingame, CA) followed by the addition of 3,39-diaminobenzidine. For negative control slides, the primary antibody was replaced by rabbit IgG, polyclonal-isotype control (Abcam, Cambridge, UK).

### RNA isolation and analysis

Gene transcript levels were quantified by real-time PCR as previously described [28]. In brief, RNA was isolated using RNeasy Mini-Kit (QIAGEN, Valencia, CA). All reactions were performed with 1 μM of forward and reverse primers along with SYBR Green I GoTaq qPCR Master Mix (Promega, Madison, WI). Primer sets were amplified using protocols previously described [28–30]. All values were normalized to a control gene such as *18S*.

### Cell culture and reagents

Murine lung epithelial 12 (MLE12) cells were obtained from ATCC (Manassas, VA) and cultured as previously described [28, 29]. Cells were plated in 6-well plates with or without bleomycin (50 μg/ml) or Akt inhibitor (1 μM). After 24 h, supernatant was collected and centrifuged to remove cellular debris and then stored at − 80 °C. Whole cell lysates were also collected to measure transcript or protein levels.

### Lentiviral shRNA generation and transduction to MLE12 cells

pLKO.1-based lentiviral *Ap3b1* shRNA constructs (RHS4533; clone ID, TRCN0000118642) were used to silence the AP3 gene in MLE12 cells in order to create cells reminiscent to those in the lungs of HPS-2 patients. Scrambled shRNAs were used as a control. Lentiviral transductions for both *Ap3b1* and scrambled control were performed as previously described [28].

### Western blot analysis

Protein concentration was determined by Pierce™ BCA assay kit (Thermo Scientific, Rockford, IL). Aliquots of protein lysates were transferred onto nitrocellulose membranes and then blocked with the Odyssey Blocking Buffer (Li-Cor Biosciences, Lincoln, NE) for 1 h at RT. This step was followed by an incubation step with a specific polyclonal rabbit primary antibody directed against MMP-2, MMP-9, Akt, phosphorylated Akt, or β-actin (Sigma-Aldrich, St. Louis, MO). Next, membranes were incubated in a solution containing a donkey anti-rabbit or anti-mouse antibody (Li-Cor Biosciences, Lincoln, NE). After three sequential washes with PBS, immunoblots were visualized using the Odyssey infrared imaging system (Li-Cor Biosciences, Lincoln, NE).

### Statistical analysis

Data are expressed as mean + SE. Differences between groups were performed using an unpaired Student’s t-test or multiple comparisons with the Bonferroni-Dunn correction. Statistical significance was achieved when *P* < 0.05 at 95% confidence interval.

## Results

### Matrix metalloproteinase activity is increased in the lungs of HPS2 mice

**Fig. 1:**
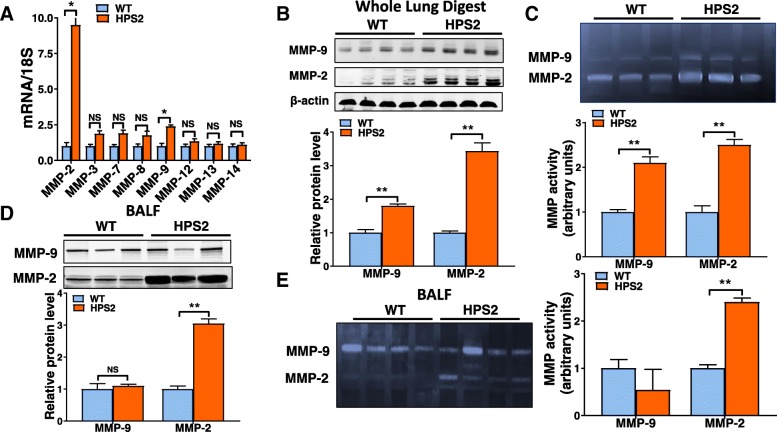
Expression and activity of matrix metalloproteinases are increased in the lungs of HPS2 mice **a**) Transcript levels for *Mmp-2, − 3, − 7, − 8, − 9, − 12, − 13* and *− 14* in age-matched control and HPS2 mouse lungs (*n* = 4 each group, *p* < 0.05 vs control). **b** Western blot for MMP-2 and MMP-9 in whole lung digests from control and HPS2 mice. **c** Gelatin zymography for MMP-2 and MMP-9 in whole lung digests from control and HPS2 mice. **d** Western blot for MMP-2 and MMP-9 in bronchoalveolar lavage fluid from control and HPS2 mice. **e** Gelatin zymography for MMP-2 and MMP-9 in bronchoalveolar lavage fluid from control and HPS2 mice. Immunoblots are representative of at least two different blots and densitometry analyses (bar graphs) are representative *n* = 5 or more mouse specimens (*p < 0.05 HPS2 vs. control). Data are expressed as mean ± SE, and statistical significance was assessed using a Student’s unpaired t test or multiple comparisons

To assess whether HPS alters MMP levels in the lung, we first performed quantitative PCR to evaluate transcript levels for several different MMPs known to be expressed in the mouse lung and which have also been linked to lung disease, including *Mmp-2, − 3, − 7, − 8, − 9, − 12* and *− 14*. As demonstrated in Fig. 1a, we found that transcript levels for each of the *Mmps* evaluated were readily detectable in the lungs of wild-type mice and that levels for most, if not all, *Mmps* were upregulated in the lungs of HPS2 mice. However, only transcript levels for *Mmp-2* and *-9* were found to be significantly increased (*p*-value < 0.05) relative to controls, and only levels of *Mmp-2* and *Mmp-9* were increased by more than 2-fold. Consistent with the marked upregulation in *Mmp-2* and *Mmp-9* expression, we found that protein levels and enzymatic activity for each of these enzymes were dramatically increased in whole lung tissue digests of HPS2 mice (Fig. 1b, c). In contrast, only levels and enzymatic activity of MMP-2 were increased in BALF (Fig. 1d, e). Altogether, these findings indicate that expression and activity of MMPs, especially the gelatinases MMP-2 and MMP-9, are increased in the lung of HPS mice.

### MMP activity is increased in the lung epithelium of HPS2 mice

**Fig. 2:**
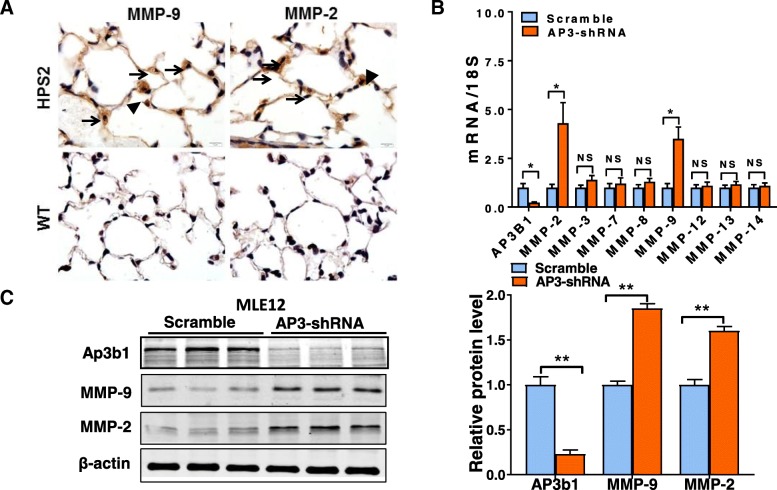
HPS2 deficiency leads to increased MMP expression in lung epithelial cells. **a** Immunohistochemical staining for MMP-2 and MMP-9 (brown staining) in the lungs of wild-type and HPS2 mice. Staining is not detected in the lungs of wild-type mice and alveolar epithelial type II cells are difficult to visualize due to normal size lamellar bodies. Expression of MMP-2 and MMP-9 appears to be increased most significantly in alveolar epithelial type II cells, as indicated by high intensity brown staining in cells containing prominent lamellar bodies (arrows). Brown staining was also evident in some alveolar macrophages (arrow heads) and in the interstitial space. **b**, **c** shRNA knockdown of the *Ap3b1* gene in MLE12 cells leads to a marked upregulation in transcript (*n* = 4, per group) and protein levels for MMP-2 and MMP-9. Immunoblot is representative of at least two different blots and densitometry analyses (bar graphs) (*n* = 5 per group, *p < 0.05, HPS2 vs. control). Data are expressed as mean ± SE, and statistical significance was assessed using a Student’s unpaired t test

Because MMPs are produced by many different cell types, we next sought to localize the expression of MMP-2 and -9 in the HPS2 mouse lung. As shown in Fig. 2a, immunostaining for MMP-2 and -9 did not detect significant protein expression in the lungs of wild-type mice. In contrast, we found that levels of both enzymes were readily detectable in the lungs of HPS2 mice and that staining was most abundant in AEC2 of the distal pulmonary epithelium, as judged by the cellular location and presence of lamellar bodies (vacuolated structures) in intensely stained cells (brown color) (Fig. 2a). Of note, lower intensity staining was also observed in other regions of HPS2 lung, including the pulmonary interstitium and alveolar macrophages, suggesting that mesenchymal cells might also contribute to elevated MMP levels in the lungs of these mice.

Because expression of MMP-2 and -9 was readily apparent in AEC2, we next sought to determine whether epithelial deficiency of the *Ap3b1* gene could by itself increase the expression of MMP enzymes. To test this, we performed shRNA knockdown of the *Ap3b1* gene in murine lung epithelial 12 (MLE12) cells, a cell line often used to model AEC2 in culture [28, 29]. Consistent with findings in vivo, we found that shRNA knockdown of *Ap3b1* readily increased MMP-2 and -9 expression, as demonstrated by a greater than 2-fold increase in transcript levels and a nearly 50% increase in protein levels for both MMP enzymes (Fig. 2b, c).

### Matrix metalloproteinase activity is increased in the lung of HPS2 mice after bleomycin

**Fig. 3:**
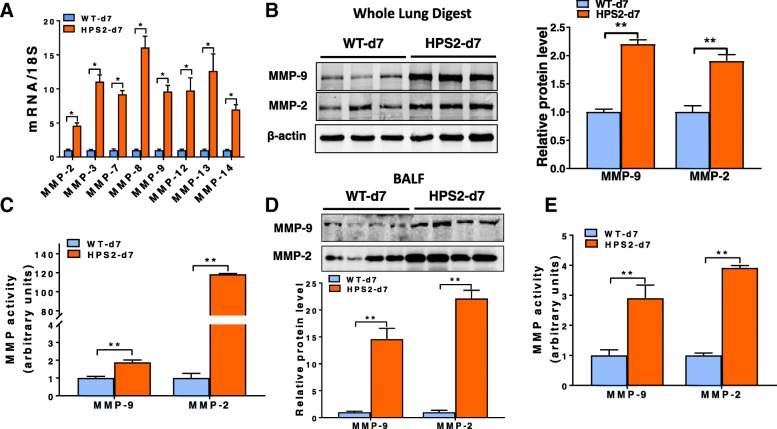
MMP levels are increased in the lung of HPS2 mice after bleomycin. **a** Transcript levels for *Mmp*-2, − 3, − 7, − 8, − 9, − 12, − 13 and − 14 in age-matched control and HPS2 lungs 7 days after bleomycin (*n* = 4 each group, *p* < 0.05 vs control). **b** Western blot for MMP-2 and MMP-9 in whole lung digests from control and HPS2 mice at 7 days after bleomycin. **c** Densitometry from gelatin zymography for MMP-2 and MMP-9 in whole lung digests from control and HPS2 mice at 7 days after bleomycin. **d** Western blot for MMP-2 and MMP-9 in bronchoalveolar lavage fluid from control and HPS2 mice at 7 days after bleomycin. **e** Densitometry from gelatin zymography for MMP-2 and MMP-9 in bronchoalveolar lavage fluid from control and HPS2 mice at 7 days after bleomycin. Immunoblots are representative of at least two different blots and densitometry analyses (bar graphs) are representative *n* = 5 or more mouse specimens (*p < 0.05, HPS2 vs control). Data are expressed as mean ± SE, and statistical significance was assessed using a Student’s unpaired t test

Expression of MMPs is known to increase in response to pro-fibrotic pulmonary insults, leading us to examine whether levels of these enzymes were further dysregulated in the HPS2 lung after pulmonary challenge. To test this, we administered a one-time, low dose (0.025 U) of bleomycin into the oropharynx of wild-type and HPS2 mice. The decision to use a low-dose of bleomycin was based on the understanding that HPS mice are exquisitely sensitive to this genotoxic insult, and that higher doses are universally fatal [17, 18, 26]. Consistent with this being a mild pulmonary insult, we found that low-dose bleomycin had little to no effect on the expression of MMPs in the lungs of wild-type mice at day 7 after injury (data not shown). In contrast, transcript levels for all of the MMPs assessed were significantly increased in the lungs of HPS2 mice relative to injured wild-type controls (Fig. 3a). Moreover, elevated transcript levels were also associated a marked upregulation in protein expression (Fig. 3b, d) and a dramatic increase in enzymatic activity for MMP-2 and -9 in whole lung lysates and BAL fluid (Fig. 3c, e).

### Elevated MMP levels are associated with an increase in Akt activation in HPS

**Fig. 4:**
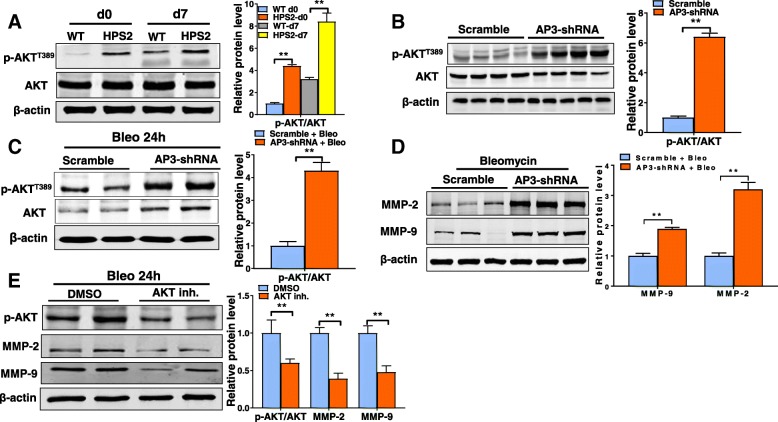
Akt activity is increased in HPS2 lung tissues. **a** Total and phosphorylated Akt levels in whole lung digests from control and HPS2 mice at baseline and at 7 days after bleomycin (left). Ratio of phosphorylated to total Akt levels (right). **b** Total and phosphorylated Akt levels in control and HPS2-like MLE12 cells at baseline (left). Ratio of phosphorylated to total Akt levels (right). **c** Total and phosphorylated Akt levels in control and HPS2-like (*Ap3b1* deficient) MLE12 cells at 24 h after bleomycin (left). Ratio of phosphorylated to total Akt levels (right) **d**) MMP-2 and -9 levels in control and HPS2-like (*Ap3b1* deficient) MLE12 cells at 24 h after bleomycin. **e**. Treatment with Akt inhibitor reduces MMP-2 and -9 levels in control and HPS2-like (*Ap3b1* deficient) MLE12 cells at 24 h after bleomycin. Immunoblots are representative of at least two different blots and densitometry analyses (bar graphs) are representative *n* = 5 or more mouse specimens (*p < 0.05, HPS2 vs control). Data are expressed as mean ± SE, and statistical significance was assessed using a Student’s unpaired t test

Since broadly inhibiting MMP activity has been associated with significant toxicity in numerous cancer studies [19], we sought to examine the effects of inhibiting an upstream regulator of MMP activity. This would also circumvent the need to simultaneously inhibit multiple MMP enzymes. Recent work has shown that MMP expression can be regulated by the enzyme Akt [21, 31, 32], leading us to hypothesize that dysregulation of Akt might contribute to altering MMP expression in the HPS lung. To test this hypothesis, we compared levels of total and phosphorylated forms of this enzyme in control and HPS tissues. Although we did not detect a significant increase in total Akt levels, the activated form of this enzyme was dramatically increased in whole lung digests of HPS2 mice at baseline and at 7 days after bleomycin. Similarly to whole lung tissues, we found that phosphorylated Akt levels were also increased in AP3 deficient lung epithelial cells (Fig. 4b) at baseline and at 24 h after bleomycin exposure (Fig. 4c) and that this associated with an upregulation in MMP-2 and -9 expression (Fig. 4d). In order to determine whether Akt regulates MMP expression, we exposed cells to a pharmacological inhibitor of Akt to examine the effects on MMP levels. As shown in Fig. 4e, we found that pharmacological inhibition of Akt significantly reduced MMP levels in bleomycin-exposed cells, supporting the notion that chronic activation of Akt contributes to elevated MMP expression in the HPS lung.

### MMP activity is increased in the lungs of HPS1 mice

**Fig. 5:**
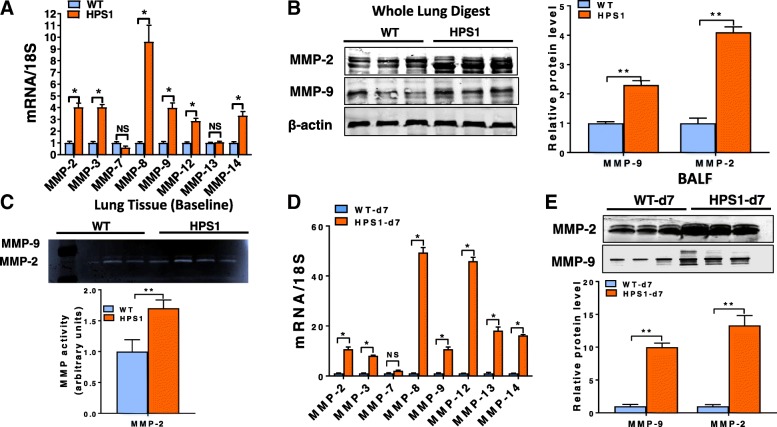
MMP levels are increased in the lungs of HPS1 mice. **a** Transcript levels for *Mmp-*2, − 3, − 8, − 9, − 12, − 13 and − 14 in age-matched control and HPS1 lungs at baseline (n = 4 each group, p < 0.05 vs control). **b** Western blot (left) for MMP-2 and MMP-9 in whole lung digests from control and HPS1 mice at baseline. **c** Gelatin zymography for MMP-2 and MMP-9 in lungs of control and HPS1 mice (top). Activity of MMP-2 but not MMP-9 was significantly increased in the lungs of HPS1 mice. **d** Transcript levels for *Mmp*-2*, − 3,-7, − 8, − 9, − 12, − 13* and *− 14* in age-matched control and HPS1 lungs 7 days after bleomycin (*n* = 4 each group, *p* < 0.05 vs control). **e** Western blot for MMP-2 and MMP-9 in whole lung digests from control and HPS1 mice at 7 days after bleomycin. Immunoblots are representative of at least two different blots and densitometry analyses (bar graphs) are representative of *n* = 5 or more mouse specimens (*p < 0.05, HPS2 vs control). Data are expressed as mean ± SE, and statistical significance was assessed using a Student’s unpaired t test or multiple comparisons

Next, to determine whether MMP expression is dysregulated in other HPS models, we measured transcript levels for various MMPs in the lungs of HPS1 mice. Strikingly, we detected a marked upregulation in transcript levels for multiple MMPs in the lungs of HPS1 mice, including *Mmp-2* and *-9* as well as the *Mmps 3, − 8, − 12* and *− 14* (Fig. 5a). Similarly to HPS2 mice, we also found that protein levels for MMP-2 and -9 were increased in whole lung digests and that MMP-2 gelatinase activity was increased in the BALF fluid relative to age-matched controls (Fig. 5b, c). Likewise, transcript levels for MMPs were also dramatically increased in the lungs of HPS1 mice after bleomycin (Fig. 5d), and this associated with elevated MMP-2 and -9 protein levels in whole lung tissue digests (data not shown) and BALF (Fig. 5e).

### MMP activity is increased in the lung of HPS patients

**Fig. 6:**
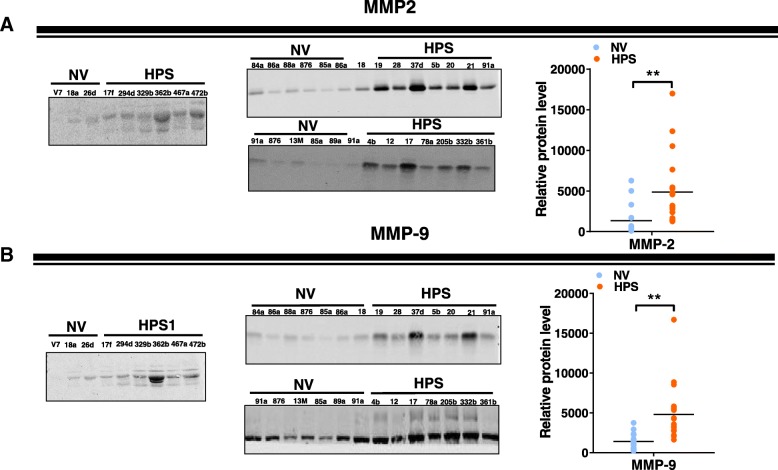
MMP-2 and MMP-9 levels are increased in bronchoalveolar lavage fluid of HPS patients. **a** Western blot for MMP-2 in bronchoalveolar lavage fluid from control and HPS patients. **b** Western blot for MMP-9 in bronchoalveolar lavage fluid from control and HPS1 patients. Dot plot depicting relative levels of MMP-2 and -9 in BAL fluid of control and HPS patients. Immunoblots are representative of at least two different blots (*p < 0.05, HPS vs control). Data are expressed as mean ± SE, and statistical significance was assessed using a Student’s unpaired t test

**Fig. 7:**
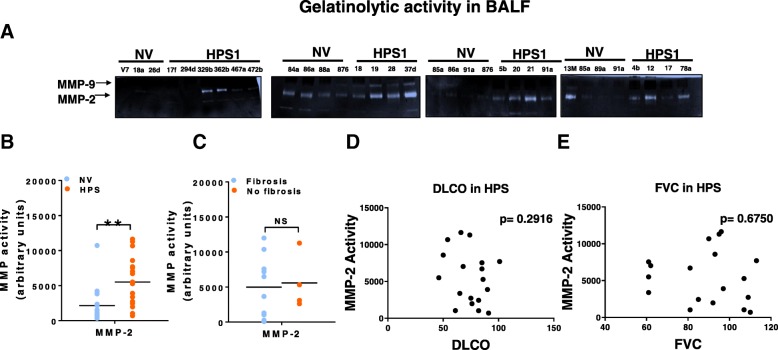
MMP activity in the bronchoalveolar lavage fluid of HPS patients. **a** Gelatin zymography for MMP-2 and -9 in the bronchoalveolar lavage fluid of control and HPS patients. **b** Dot plots depicting levels of MMP-2 in BAL fluid of control patients and HPS patients with and without known lung fibrosis. **c**, **d**, **e** Dot plot depicting the relationship between MMP-2 activity and diffusion capacity of lung for carbon monoxide and forced vital capacity in HPS patients, respectively

Finally, to determine whether findings in mouse models were relevant to human disease, we assessed whether levels or activity of MMPs were altered in the lungs of HPS patients. As shown in Fig. 6, we found that protein levels for both MMP-2 and -9 were significantly increased in BALF of HPS patients relative to controls. Moreover, this associated with a significant upregulation in MMP-2 activity (Fig. 7a), although MMP-9 activity did not significantly differ between control and HPS patients (Fig. 7b). Interestingly, neither levels of MMP-2 and -9 nor activity of MMP-2 associated with the presence or absence of fibrosis or measures of lung function, such as diffusing for carbon monoxide or forced vital capacity (Fig. 7c, d).

## Discussion

Mutations linked to HPS have been well-characterized but how these mutations ultimately lead to the development of pulmonary fibrosis remains unknown. In this study, we demonstrated that mutations in two different HPS genes lead to a similar upregulation in the expression and activity of MMPs in the mouse lung. Furthermore, we found that these changes occurred before the onset of lung fibrosis and were magnified after instillation of a low-dose of bleomycin into the lung. Additionally, we uncovered that increased MMP levels were also observed in the BALF of subjects with HPS, and that these changes, like in mice, were detectable in some individuals with no evidence of lung disease. Taken together, these findings indicate that HPS-related genes are important for regulating MMPs in the lung, and suggest that altered MMP expression due to HPS mutations might contribute to lung disrepair and fibrotic remodeling.

A large number of MMP genes exist within the mouse and human genome [7]. For example, at least 23 different MMP genes have been identified within the mouse genome and even more are believed to exist in humans. In this study, we used a targeted approach to examine MMP levels in the lung, measuring only those enzymes known to be expressed in mouse or human respiratory tissues [7]. Using this targeted approach, we uncovered the upregulation of multiple MMPs in the lungs of HPS mice. Interestingly, although transcript levels for MMP-2 and -9 were most significantly increased in HPS2 mice, we detected a broad upregulation in many different MMP transcripts in the lungs of HPS1 mice, including a greater than 2-fold increase in levels for MMP-2, − 3, − 8, − 9,-12 and − 14. These findings suggest the intriguing possibility that HPS is a heterogeneous group of disorders, and that pathological processes contributing to the development of pulmonary fibrosis might differ among individuals carrying different HPS mutations.

Although MMPs are produced by many different cell types in the lung, our findings suggest that epithelial cells are an important source of MMP production in the HPS lung. This was demonstrated by the marked increase in MMP-2 and -9 expression in the distal pulmonary epithelium of HPS mice and by the striking increase in MMP expression in cultured lung epithelial cells after knocking down the *Ap3b1* gene. Importantly, these findings support the current paradigm in both the IPF and HPS fields that epithelial dysfunction and abnormal crosstalk of epithelial cells with mesenchymal cells contributes to the development of disease [26, 33–36].

Akt is emerging as an important pathogenic player in pulmonary fibrosis [20, 21]. For example, Akt activation has been tightly linked to growth, survival and differentiation of activated lung fibroblasts and inhibition of this enzyme has been shown to reduce experimentally-induced pulmonary fibrosis in mice [20, 31, 32, 37]. In addition, Akt activity is also known to be increased in the lung epithelium of mice with pulmonary fibrosis as well as the lung epithelium of patients with IPF [21]. In epithelial cells, chronic activation of Akt is thought to prime the lung for injury through reducing the expression of cell-cell junctional proteins and impairing epithelial barrier protection. Consistent with this, our study provides further support for the concept that hyperactivation of Akt contributes to the development of pulmonary fibrosis in HPS and that targeting this kinase might be a strategy for preventing or treating this disease.

Classically, levels of MMP enzymes are relatively low in healthy tissues and dramatically increase in response to tissue insults or disease states [6, 7, 10, 38]. However, to our surprise, we found that levels of MMP enzymes were markedly increased in the HPS lung under homeostatic conditions. This included the lungs of HPS1 and HPS2 mice as well as the lungs of HPS subjects without evidence of disease. Interestingly, we did not observe a relationship between MMP activity and lung function (diffusion capacity or forced vital capacity) in our cohort of HPS patients, suggesting the intriguing possibility that chronically elevated MMP levels might contribute to the onset rather than the progression of disease. In this scenario, we wonder whether elevated MMP levels might either prime the lung for injury or make it susceptible to disrepair. Importantly, our study investigated only a limited number of MMPs, leaving the possibility that associations might be identified between other MMPs and disease activity.

Our study has several notable limitations. First, we focused only on HPS mouse models known to be susceptible to pulmonary fibrosis, preventing us from determining whether MMP levels are also dysregulated in the lungs of mice harboring other HPS mutations. Second, we measured activity of only MMP-2 and -9 in our samples, which does not allow us to comment on whether activity of other MMPs are elevated in the HPS lung. Third, our study measured MMP levels in a small cohort of patients and our population included a mixture of different HPS populations, including two individuals with HPS4 mutations. Finally, although bleomycin is considered the gold-standard pulmonary fibrosis model, it has significant limitations, including the fact that inflammation drives much of the fibrotic remodeling and tissue remodeling is completely reversible to this insult. Despite these shortcomings, the observation that similar MMPs are dysregulated in both the HPS mouse and human lung supports the validity of using this model in our investigations.

## Conclusions

In summary, our findings indicate that MMP levels are upregulated in the HPS lung and that these changes precede the development of pulmonary fibrosis. Future studies determining whether elevated MMP levels assist in the diagnosis of HPS will be important. Furthermore, understanding whether dysregulation of MMPs contributes to the onset or progression of HPS lung disease will ultimately be important for advancing understanding of disease and laying the foundation for new and more effective treatments.

## Additional file

Additional file 1: Clinical Demographics of HPS and control subjects. (PDF 133 kb)

## Data Availability

The datasets used and/or analyzed during the current study are available from the corresponding author on reasonable request.

## Abbreviations

AKT: Protein Kinase B
Ap3b1: Adaptor protein complex 3-subunit beta-1
BALF: Bronchoalvelolar lavage fluid
Bloc3: Biogenesis of lysosome-related organelles complexes 3
HPS: Hermansky-Pudlak Syndrome
IPF: Idiopathic Pulmonary Fibrosis
MMP: Matrix Metalloproteinase
PI3K: Phosphatidylinositol 3-kinase
