# Supplementary material for: Matrix metalloproteinase activity in the lung is increased in Hermansky-Pudlak syndrome
Source: Orphanet J Rare Dis. 2019 Jul 4;14:162. doi: 10.1186/s13023-019-1143-0 (PMC6610946; doi:10.1186/s13023-019-1143-0)
Supplement: Supplementary file 1 — Clinical Demographics of HPS and control subjects. (PDF 133 kb) [file 13023_2019_1143_MOESM1_ESM.pdf]

Pt Code	Genotype	Age	Gender	Fibrosis	FVC	DLCO	HRCT ILD	Honeycombing	Cysts	GGO
V7	NV	56	M	no	106	103	no	no	no	no
18a	NV	19	F	no	103	101		no	no	no
26d	NV	32	M	no	103	109	no	no	no	no
17f	HPS-1	49	F	yes	110	91	yes	no	no	no
294d	HPS-1	43	F	yes	93	50	yes	no	yes	no
329b	HPS-1	36	F	yes	62	68	yes	no	no	yes
362b	HPS-1	55	M	yes	92	76	yes	no	no	no
467a	HPS-1	62	M	yes	81	82	yes	no	no	no
472b	HPS-1	36	F	yes	85	82	yes	no	no	no
84a	NV	29	M	no	100	114		no	no	no
86a	NV	26	F	no	90	84		no	no	no
88a	NV	45	M	no	104	96		no	no	no
876	NV	41	M	no	125	112		no	no	no
18	NV	25	F	no	113	101	no	no	no	no
19	HPS-1	38	F	yes	61	85	yes	no	no	yes
28	HPS-1	44	M	yes	96	66	yes	yes	no	no
37d	HPS-4	32	F	no	61	65	no	no	no	no
85a	NV	47	F	no	123	101		no	no	no
86a	NV	26	F	no	90	84		no	no	no
91a	NV	29	M	no	82	93		no	no	no
876	NV	41	M	no	125	112		no	no	no
5b	HPS-1	37	F	no	107	86	no	no	no	no
20	HPS-1	37	M	no	95	74	no	no	no	no
21	HPS-4	62	F	yes	109	75	yes	yes	no	no
91a	HPS-3	20	M	no	97	90	no	no	no	no
13M	NV	51	M	no	92	77	no	no	no	no
85a	NV	47	F	no	123	101		no	no	no
89a	NV	22	M	no	108	91		no	no	no
91a	NV	29	M	no	82	93		no	no	no
4b	HPS-1	37	F	yes	90	54	yes	no	no	yes
12	HPS-1	37	F	yes	107	61	yes	no	yes	no
17	NV	43	F	no	81	85	no	no	no	no
78a	HPS-1	49	M	yes	61	46	yes	yes	no	yes
